# Design of Bilayer Crescent Chiral Metasurfaces for Enhanced Chiroptical Response

**DOI:** 10.3390/s25030915

**Published:** 2025-02-03

**Authors:** Semere A. Asefa, Myeongsu Seong, Dasol Lee

**Affiliations:** 1 Department of Biomedical Engineering, Yonsei University, Wonju 26493, Republic of Korea; semrelbu@yonsei.ac.kr; 2 Department of Mechatronics and Robotics, School of Advanced Technology, Xi’an Jiaotong-Liverpool University, Suzhou 215123, China

**Keywords:** chiral metasurface, chiroptical response, metasurface, metamaterials, chiral sensing, circular dichroism, chirality, layered chiral metasurface, tunable chiral metasurface, crescent chiral metasurface

## Abstract

Chiral metasurfaces exploit structural asymmetry to control circular polarized light, presenting new possibilities for the design of optical devices, specifically in the dynamic control of light and enhanced optical sensing fields. This study employed theoretical and computational methods to examine the chiroptical properties of a bilayer crescent chiral metasurface, demonstrating the effect of the angle of rotation on the chiroptical response. We particularly investigated the changes in transmittance, electric field distribution, and circular dichroism (CD) across various rotation angles. The crescent chiral metasurface demonstrated the maximum CD and showed significant control over the CD and electric field distribution across different rotation angles in the near-infrared region. The highest CD value was observed at a 23° rotation angle, where the chiroptical response reached its maximum. In addition, the left circular polarized light showed a stronger intensity of the electric field along the crescent metasurface edge relative to the right circular polarized light, underscoring the significant difference in the intensity and field localization. It was also shown that the configuration with a 2 by 2-unit cell, compared with a single-unit cell, exhibited significantly enhanced CD, thus underlining the importance of the unit cell arrangement in optimizing the chiroptical properties of metasurfaces for advanced photonic applications. These results prove that the 2 by 2 bilayer crescent chiral metasurface can be tailored to a fine degree for specific applications such as improved biosensing, enhanced optical communications, and precise polarization control by optimizing the configuration. The insight presented by this theoretical and computational study will contribute to the broad understanding of chiroptical phenomena as well as pave the way for potential applications in developing advanced optical devices with tuned chiroptical interactions.

## 1. Introduction

Chirality is observed on scales ranging from the biological building blocks of nature including DNA, sugar, and amino acids to its pinnacle, the spirals of galaxies. Chiral materials, which have left- or right-handedness, have revolutionized the state-of-the-art of present-day optics and photonics [1,2]. Engineering these materials has given rise to special chiroptical phenomena including circular dichroism (CD) and optical rotation. CD is the absorption difference between the left circular polarized (LCP) and right circular polarized (RCP) [2]. Chiroptical response or chiroptical activity, which is characterized by the CD, refers to the distinct optical property of chiral materials due to the difference in absorption for the LCP and RCP, which enables a wide range of applications in chiral sensing [2,3,4,5,6,7], metalenses [3,8,9,10,11], imaging [12,13,14], and telecommunication systems [15,16,17,18].

Chiral metasurfaces are 2D chiroptical metamaterials with subwavelength unit cells to improve chiroptical responses [2,19,20,21,22,23,24,25,26]. In general, chiral metasurfaces can be divided into two categories: plasmonic and dielectric. Plasmonic metasurfaces utilize localized surface plasmon resonances and provide strong light confinement and field enhancement, thus enabling efficient light manipulation that can potentially enhance chiral effects with respect to their dielectric counterparts [14,17,26,27,28]. The ohmic losses in plasmonic chiral metasurface can be carefully minimized through design; hence, plasmonic metasurfaces are a very promising platform for achieving strong chiral responses. In contrast, dielectric metasurfaces rely on geometric features combined with a high refractive index contrast to minimize ohmic losses and thus provide a suitable approach for applications that have high transmission and efficiency requirements [27,28].

Chiral metasurfaces are designed to manipulate the asymmetric characteristics of materials, enabling them to interact with LCP and RCP light differently. This potential is significant for applications demanding control over polarization including highly selective light filters, advanced optical sensors, biosensing, and novel imaging systems [1,6,19,29,30]. Leveraging CD and other chiroptical effects, these metasurfaces provide a robust platform for advancing the next-generation optical devices. The capability of chiral metasurfaces to transform fields from biomedical diagnostics to quantum computing demonstrates a profound convergence between wave engineering and material science [1,2,6,19,29,30,31,32].

The chiroptical response of the chiral metasurface hinges on the manipulation of geometric parameters [2]. In contrast to single layer metasurfaces, multi-layer metasurfaces composed of noble metals enable us to design and control the optical responses [2,33,34]. The multi-layer chiral metasurface’s chiroptical response depends on the number of layers, inter-layer distance, and the rotation angle between the meta-atom layers [15,34,35,36]. For example, multi-layer nano-rods, split-ring resonators, and cylindrical oligomers have strong coupling and chiroptical response depending on the rotation angles between the layers of meta-atoms and inter-layer distance [10,15,34,37,38]. Thus, the chiroptical response can be tailored by the geometric parameters as the chiroptical response is related to the electromagnetic interactions in between the adjacent layers [2,34,39].

The advancements in chiral metasurface design have centered on maximizing the chiroptical response by finely adjusting the subwavelength structure. Considerable progress has been made in the development of different chiral metasurfaces with the aim of enhancing the chiroptical response [2]. By carefully designing the geometry, arrangement, and composition of the meta-atoms within the metasurface, researchers have achieved impressive results in manipulating the polarization state and CD of transmitted or scattered light [29,30,40]. The chiroptical response can also be improved by the bound states in the continuum (BICs), which can support high-Q resonances with highly localized light and minimal radiation losses [41,42]. Robust circular polarizations, together with sharp spectral selectivity, have been realized in the symmetrical-breaking and coupling effects of BIC-based metasurfaces for the manipulation of chiral waves. The introduction of a magneto-optical material amplifies chirality, enabling tunable circular dichroism and polarization control via an external magnetic field, hence further extending the functionalities of 2D metamaterials [43]. These advancements have led to remarkable applications such as chiral sensing, enantioselective catalysis, and chiral light–matter interactions [29,30].

Conversely, despite the advances made in this field, several research gaps remain. Most of the existing BIC-based designs suffer from the sensitivity of geometric perturbations and narrow operational bandwidths. Therefore, there is a need to explore modern design strategies to further improve the chiroptical response of chiral metasurfaces [2,29,40]. Enhancing the chirality, bandwidth, efficiency, and polarization control of metasurfaces will be crucial in enabling their widespread implementation in wider applications. Moreover, a deeper understanding of the fundamental mechanisms governing chiral light–matter interactions is key to unlocking the full potential of chiral metasurfaces [2,33,34,39].

As an endeavor to fill the gaps, we studied a bilayer crescent shaped asymmetric metasurface to improve the chiroptical response. A bilayer crescent metasurface is a two-layer structure made up of stacked layers that are composed of crescent-shaped meta-atoms to enhance chiroptical interactions due to the interlayer coupling effects. Initially, we demonstrate the geometric design and the theoretical basis of the 2 by 2-unit cell of the crescent chiral metasurface. Subsequently, we present a single-unit cell of the bilayer crescent metasurface to evaluate the effect of the unit-cell configurations on the chiroptical response. We measured the transmittance of the 2 by 2- and single-unit cell of the crescent metasurface, then proceeded to examine the CD for the LCP and RCP light in the NIR, assessing the effect of the rotation angle on the chiroptical response. Further analysis examined the electric field distribution along different rotation angles. This study deepens our understanding of the interaction between the bilayer crescent chiral metasurface and the chiroptical response, furthering profound insights into the dynamics concerned with the transmittance and distribution of the CD electric field, as dictated by the geometric parameters.

## 2. Methodology

The study encompassed a comprehensive analysis of the 2 by 2-unit cell crescent chiral metasurface to enhance the chiroptical response. The transmittance, CD, and electric field distribution analysis were conducted across different parameters such as the rotation angle (α) and inter-layer space (d). To optimize the design of the crescent chiral metasurface, a single-unit cell was also introduced under the same conditions. The methodology employed in this study involved the utilization of numerical simulations through the COMSOL Multiphysics software to design and analyze the metasurface.

### 2.1. Geometric Design and Theoretical Basis

#### Geometric Design

**Figure 1:**
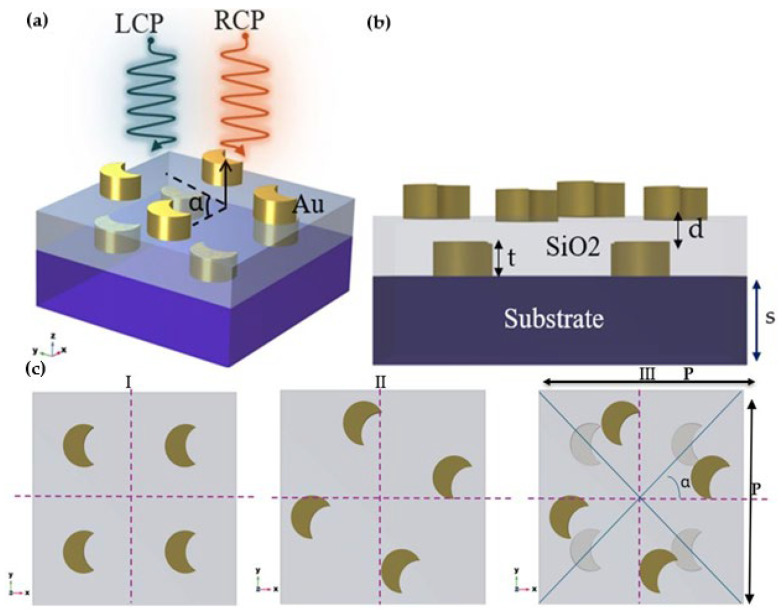
The geometry of the metasurface. (**a**) The crescent chiral metasurface meta-atom arrangements. (**b**) Side view of the bilayer crescent chiral metasurface, where d is the inter-layer distance, t is the thickness of the meta-atom, and s = 100 nm is the thickness of the substrate. (**c**) The arrangement of the chiral metasurface top view, where (Ⅰ) is the bottom layer of the chiral metasurface, (Ⅱ) is the top-layer at a rotation angle of α, and (Ⅲ) is the inter-layer twist: the relative rotational alignment between the two layers, where P = 500 nm is the width and length of the chiral metasurface.

The crescent structure of the metasurface has a unique geometric asymmetry and provides robust interaction with LCP and RCP light. The crescent geometry provides broad design flexibility to control across the resonance frequency. Such properties make the crescent metasurface ideal for applications in polarization manipulation, optical sensing, and photonic devices, with a new capability to manipulate light at the level of the nanoscale. The geometric design of the crescent chiral metasurface involved thoroughly defining the geometric parameters. The rotation angle (α), inter-layer distance (d), and unit cell arrangement of the crescent chiral metasurface were optimized to enhance their interaction with the CPL. The crescent bilayer structure consisted of two layers, each with four crescent meta-atoms (Figure 1a). Gold was used as the meta-atom due to the potent surface plasmon resonance, and silicon dioxide for the inter-layer space due to its high transmittance in the NIR. The second layer was positioned at a rotation angel α and distance ‘d’ from the first layer. The crescent meta-atom had an outer radius of 200 nm, inner of 100 nm, and a thickness of 100 nm. The rotation angle and inter-layer distance were optimized to maximize the circular dichroism and electric field distribution. The material properties of gold were allocated using the modified Drude model [44].

### 2.2. Theoretical Basis of the Crescent Chiral Metasurface

#### 2.2.1. Jones Matrix and Circular Dichroism

Chirality is the phenomenon in which a material cannot overlap with its mirror through rotation or translation. Circular dichroism is the absorption difference between the left and right circular polarized light and is often calculated mathematically by the 2×2 Jones matrix, which describes the polarization properties of optical materials.

The crescent structure of the meta-atoms is asymmetric and lacks rotational symmetry, which ensures that it responds differently to the LCP and RCP light. The transmission matrices from the linear to the circular polarization are given below [2]:

(1) $$T\;=\left[\begin{array}{cc} t_{++} & t_{+-}\\ t_{-+} & t_{--} \end{array}\right]=\frac{1}{2}\left[\begin{array}{cc} t_{xx}+t_{yy}+i(t_{xy}-t_{yx}) & t_{xx}-t_{yy}-i(t_{xy}+t_{yx})\\ t_{xx}-t_{yy}+i(t_{xy}+t_{yx}) & t_{xx}+t_{yy}-i(t_{xy}-t_{yx}) \end{array}\right]\;={\;M}^{-1}TM,$$

where $t_{xy}$ is the transmission coefficient for y-polarized incident light to x-polarized transmitted light. M = $\frac{1}{\sqrt{2}}\left(\begin{array}{cc} 1 & 1\\ i & -i \end{array}\right)$ is the Cartesian to circular transformation matrix, and $t_{+-}$ represents LCP incidence and RCP transmission. Transformation is thus important to understand the chiral nature of the crescent chiral metasurface. Therefore, the conversion of linear to circular polarization captures the chiroptical characteristics in the crescent chiral metasurface. The transmissions of the co-polarized light are given below [45,46]:

(2) $$T_{RCP}={\left|t_{++}\right|}^{2}$$

(3) $$T_{LCP}={\left|t_{--}\right|}^{2}$$

where T_LCP and T_RCP are the co-polarized transmittances for the LCP and RCP light, respectively. CD is the absorption difference between the LCP and RCP light:

CD = A_LCP_ − A_RCP_ (4)

where A_LCP_ and A_RCP_ represent the absorption of the LCP and RCP light, respectively.

#### 2.2.2. Expected Outcomes and Theoretical Predictions

The theoretical predictions for the crescent bilayer chiral metasurface indicate discrete transmission spectra and CD value, characterized by a sharp resonance and major difference between the RCP and LCP light interactions. The expected electric field distribution pattern showed sections of enhanced and localized fields, specifically at the edge of the crescent structure. The localized field distribution is imperative to attain a high CD value. Theoretical studies have shown the optimization of such layered chiral structures by parameters such as rotation angle, inter-layer distance, and arrangements for an improved chiroptical response to customize the metasurface for specific applications in biosensing and photonic devices [2].

## 3. Results

### 3.1. Transmission Spectra

Chiral metasurfaces, with their exceptional quality to respond differently to RCP and LCP light, have gained substantial attention for their feasible applications in photonics, biosensing, and optics [1,6,19,29,30]. Transmittance in the crescent chiral metasurface is important since it provides information on how they manipulate the incident light. Co-polarized transmittance describes the light that has passed and retains the original polarization, while cross-polarized transmittance refers to light that has changed its polarization upon transmission [19,47]. This distinction is important when examining chiral metasurfaces, where the chiroptical properties change depending on the incident light’s polarization. In this paper, we employed co-polarized transmittance to study the chiroptical response of the metasurface. The effectiveness and efficiency of the chiral metasurface in practical applications including advancing communication devices, improving the sensitivity of biosensors, and developing advanced selective optical filters can be scrutinized by the transmittance value of the chiral metasurface [2,19,47].

**Figure 2:**
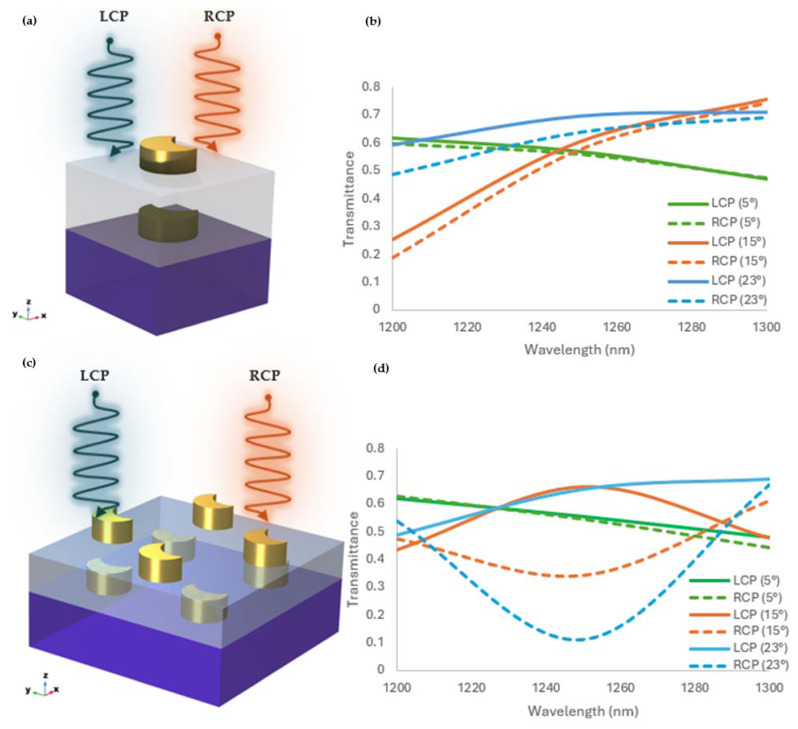
(**a**) Schematic of the single unit cell arrangement. (**b**) the transmittance of LCP light and RCP light, for the single unit cell at 5°, 15°, and 23° rotation angles. (**c**) The schematics of the 2 by 2-unit cell ar-rangement. (**d**) the transmittance of the LCP and RCP light, for the 2 by 2-unit cell at 5°, 15°, and 23° rotation angles.

In the NIR region, the transmittance of the crescent bilayer chiral metasurface was evaluated for various rotation angles (5°, 15°, and 23°). The main purpose of this assessment was to understand the effect of the geometric rotation on the crescent metasurface’s optical properties. The rotation angle between the top and bottom layer of meta-atoms is critical because it dictates the coupling effects between the layers, which in turn influence the overall chiroptical response of the metasurface. The obtained transmittance values indicate the metasurface’s ability to selectively transmit light of specific wavelengths (Figure 2b,d). Notably, it can be seen that LCP and RCP light possessed different transmittance values, reflecting troughs and peaks belonging to the resonance modes of the crescent chiral metasurface for the 2 by 2-unit cell (Figure 2d). This asymmetry in transmittance indicates the metasurface’s strong chiroptical response, where it exhibits different transmission behavior depending on the handedness of the incident light. The transmittance for the 23° rotation angle showed a unique response compared with the other rotation angles, showing a strong dependence of the crescent bilayer chiral metasurface on the rotational arrangement (Figure 2).

The unit cell arrangement of the chiral metasurface’s meta-atom can affect the chiroptical response [2,48,49]. Comparing different unit cell arrangements, such as single-unit cell and 2 by 2-unit-cell arrangement, provided us with the optimal design for the enhanced chiroptical response. Thus, we evaluated a single-unit cell (Figure 2a) and 2 by 2-unit cell arrangement (Figure 2c) with similar properties for optimizing the design. The transmittance investigation indicated significant differences in the response of the single-unit cell and the 2 by 2-unit cell layouts to LCP and RCP light. As shown in Figure 2b, the single-unit cell had a relatively low transmittance for LCP and no fluctuation in transmittance between LCP and RCP. This indicates a relatively weaker chiral interaction inside the single-unit cell structure. In contrast, the 2 by 2-unit cell layout had substantially larger transmittance changes between the LCP and RCP light, indicating a strong chiroptical response (Figure 2d). The increased transmittance variation in the 2 by 2-unit cell demonstrated enhanced electromagnetic coupling, increased effective asymmetry, and collective resonance of multi-unit cell arrangements. These findings clearly show that employing multi-unit cell topologies in advanced chiral metasurfaces improves the transmittance difference and strengthens chiral interactions.

### 3.2. Circular Dichroism (CD)

#### 3.2.1. Description and Significance of CD

Circular dichroism measures the differential absorption of RCP and LCP light by chiral structures, and it is important in outlining the chiroptical response of chiral metasurfaces [2]. CD is not just an evaluation of the optical behavior of the metasurface; it is a clear signal of the asymmetry in the metasurface’s interaction with polarized light, showing the chiroptical properties of the metasurface structure. The spectral position and magnitude of the CD value offers comprehensive insights into the geometric conformation and electronic transitions in the metasurface. Thus, analyzing the CD is important for understanding and enhancing the functionality and design of chiral metasurfaces in high-level optical applications. This parameter is crucial for many applications, ranging from the chirality detection of enantiomers in chemical analysis to the enhancement of photonic device efficiency, where precise control of the polarization of light is paramount [2,6,19,29,30].

#### 3.2.2. CD Analysis

**Figure 3:**
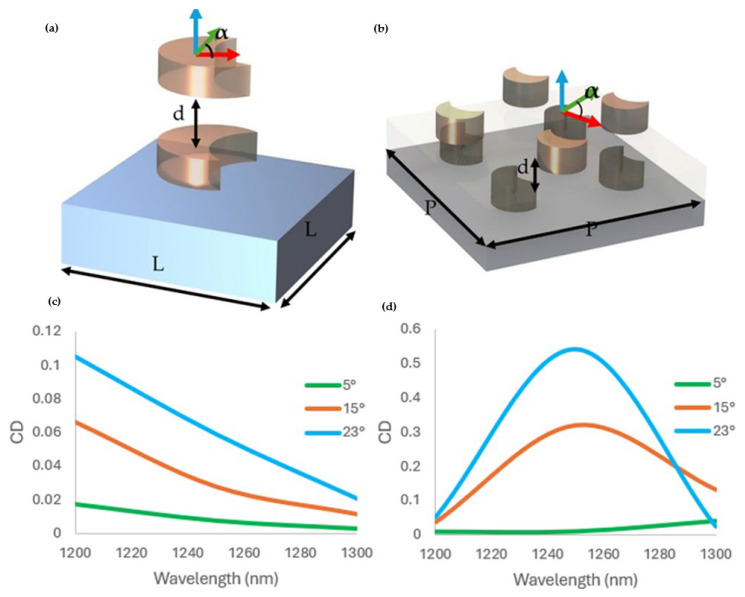
(**a**) Optimized geometric parameters of the single-unit cell arrangement, with L = 400 nm and d = 100 nm at the different rotation angles of (α) of 5°, 15°, and 23°. (**b**) The orientation of the 2 by 2-unit cell with P = 500 nm and d = 100 nm at the different rotation angles of 5°, 15°, and 23°, respectively. (**c**) Peak CD value of the single-unit cell crescent chiral metasurface along the 5°, 15°, and 23° rotation angles. (**d**) Peak CD values of the 2 by 2-unit cell crescent chiral metasurface at the 5°, 15°, and 23° rotation angles.

As shown in Figure 3c,d, the CD value exhibited eminent peaks in the NIR, ascribed to the distinctive configuration of the bilayer crescent metasurface. The highest CD obtained was in the NIR, suggesting strong chirality, as shown in Figure 3d.

To review the relation between the geometric parameters and the CD value, we examined the rotation angle effect on the maximum CD. As shown in Figure 3d, we examined the CD value of the 2 by 2-unit cell at different rotation angles, specifically the 5°, 15°, and 23° counterclockwise (CCW) rotations of the top layer with respect to the bottom layer, while keeping the other parameters constant. The CD value ranged from 0 to 0.56, as shown in the Figure 3d, and the maximum CD value of ≈0.56 was obtained at the resonance frequency with a rotational angle of 23°. This peak value was linked to the interaction of the inter-layer meta-atom and the location of the crescent meta-atoms. At the resonant frequency, the CD value was directly proportional to the rotation angle; as the rotation angle increased from 5° to 23°, the CD value increased from 0.012 to 0.56 (Figure 3d). Thus, by tuning the geometric parameters, the chiroptical response of the crescent chiral metasurface can be improved to obtain a higher CD value. Compared with symmetric structures, such as spherical or cylindrical metasurfaces, the arrangement of the crescent structure exhibited a better chiroptical response [37,39]. While the C4 symmetric structures like cylindrical meta-atoms showed relatively minimal CD as a result of their symmetrical interactions, the crescent chiral metasurface broke the rotational symmetry, leading to the maximum chiroptical response. The response obtained shows that bilayer crescent metasurfaces are efficient in producing better chirality, making them suitable for polarization control and biosensing applications.

The chiroptical response of metasurfaces depends on the phase through geometric and dynamic phases. Such a phase depends on the meta-atom orientation and dimensionality to enable the selective-phase responses of spin states. It shows that both the meta-atom arrangement and number of unit cells will affect the CD response [48]. Under similar conditions, the CD responses of the 2 by 2-unit cell consistently produced higher CD values than the single-unit cell arrangements, as shown in Figure 3c,d. Notably, at a rotation angle of 23°, the 2 by 2 structure reached a maximum CD value of 0.56, but the single unit-cell configuration produced a lower CD value of 0.1. The CD for the single-unit cell was 0.1 at 23° of rotation and 0.06 at 15°, which were much lower than the corresponding values of the 2 by 2-unit cell crescent chiral metasurface (Figure 3c,d). The 2 by 2-unit cell’s constant superiority across various rotation angles emphasizes the importance of multi-unit cell arrangements in maximizing the metasurface’s chiroptical response and couplings.

**Figure 4:**
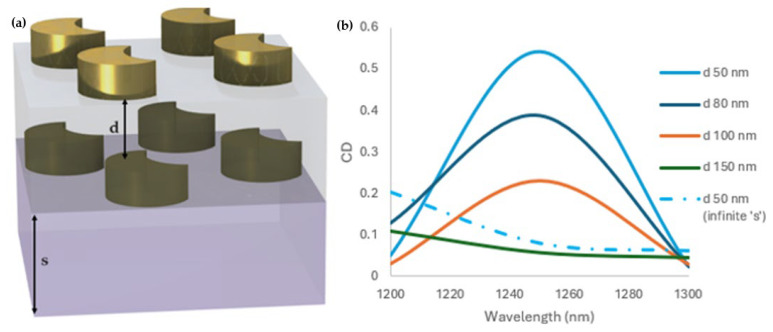
(**a**) The schematics of the 2 by 2-unit cell arrangement highlighting the inter-layer distance ‘d’ and substrate thickness s = 200 nm. (**b**) Simulated CD for a finite substrate thickness (s = 200 nm) at different inter-layer distances of d = 50 nm, 80 nm, 100 nm, and 150 nm. The hidden line in the legend represents the CD response for an infinite substrate and d = 50 nm. These results therefore signify that critical tuning by changing the substrate and inter-layer distance determines the modulated chiral property of the metasurface.

The effect of other geometric parameters on the chiroptical response was also carefully explored, with a particular emphasis on the impact of interlayer distance (d) (Figure 4a). As the inter-layer distance varied, the CD response varied (Figure 4b). The maximum CD value of the 2 by 2-unit cell decreased from 0.56 to 0.11 as the inter-layer distance increased from d = 50 nm to d = 150 nm, as shown in Figure 4b. The results emphasize the importance of fine geometric tailoring in maximizing the chiroptical properties of metasurfaces for advanced optical applications.

Hence, the chiroptical response is strong, depending on the rotation angle and interlayer distance. The chirality enhanced with an increased rotation angle ranging from 5° to 23° (Figure 3), but decreased with an increasing interlayer distance from 50 nm to 150 nm (Figure 4b). These prove that the parameters play a crucial role in optimizing the chiroptical response of a metasurface. In addition to the inter-layer distance and rotation angle, the substrate thickness (s) also significantly modulated the chiroptical response. A finite substrate enhanced the CD response due to optical effects such as interference and reflection, which amplified the local field distribution. On the other hand, an infinite substrate reduced the CD response drastically, hence showing the importance of the substrate thickness in achieving the observed chirality (Figure 4b).

### 3.3. Electric Field Distribution

**Figure 5:**
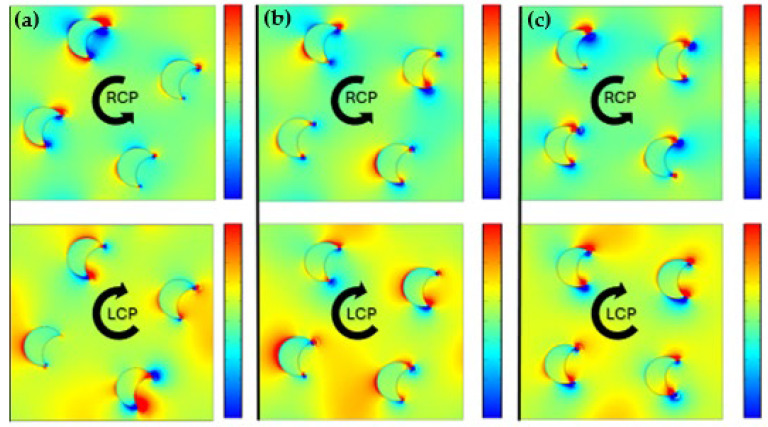
The electric field distribution for the RCP (ERCP) and LCP (ELCP) light, at different rotation angles: (**a**) 5°; (**b**) 15°; (**c**) 23°.

The electric field distribution assessment imparted clarity into the operations behind the improved chirality in the 2 by 2 bilayer crescent chiral metasurface. Accordingly, the electric field distribution along the different rotation angles corresponded to the variation in intensity occurring in the field coupling between the meta-atom layers, caused by changes in the rotation angle. This paper, therefore, explored the contribution of inter-layer rotation angles to further improve the field coupling for the optimization of chiroptical response in the metasurface, moving forward from the previous geometrical parameter investigations of shape, spacing, and orientation. By evaluating the field distribution at different rotation angles in between the layers, we were able to interpret the effect of geometric parameters on the CD and determine the finest design. The field distribution for the RCP and LCP light at the maximum chiroptical response showed the critical areas of field enhancement and localization, as shown in Figure 5c.

At the maximum chirality, the field distribution for the RCP light demonstrated a substantial concentration of the electric field at the tips and edges of the crescent structure, as shown in Figure 5c. These were the areas where the plasmonic resonance was extremely exciting, resulting in improved interactions with the incident CPL. The crescent bilayer geometry of the metasurface prompted the field to localize distinctively for the RCP and LCP light (Figure 5c).

The electric field distribution analysis demonstrated higher coupling in the LCP than RCP, as shown in Figure 5. Under the optimal chiroptical response conditions (α = 23, t = 100 nm, and d = 50 nm), the crescent chiral metasurface showed significantly increased and more localized electric field intensities, as shown in Figure 5c. This was pronounced at the edges of the crescent shapes, where the field concentration was high. Enhanced localization of fields in the meta-atoms enabled stronger interactions with the incident CPL, thus increasing the chirality. These findings show that the crescent chiral metasurface not only improved the overall field distribution, but also optimized the electromagnetic interactions required for successful chiroptical applications. This improved electric field distribution is critical for achieving increased sensitivity and performance in practical devices that use chiral metasurfaces. When the rotation angle varied from 5° to 23°, the electric field localization at the crescent tip grew, with the electric field concentration increment in regions that enhanced the chiroptical response as shown in Figure 5.

Since the chiroptical response increased with the increment in the rotation angle from 5° to 23°, the difference in the electric field of the LCP (ELCP) and the electric field of the RCP (ERCP) also became more pronounced across the meta-atom structure. Particularly, the ELCP distribution at 23° CCW showed a more pronounced and localized field compared with that at 5° (Figure 5a,b). Likewise, the ELCP distribution at 15° demonstrated a better field intensity and localization than that at 5°, as shown in Figure 5b,c. At lower rotation angles, the overlap of fields between layers was less, leading to weak plasmonic coupling and lower chirality. In contrast, for an increased rotation angle of 23°, the orientation of the bilayer crescent chiral metasurface served to enhance the near-field coupling, leading to intense electric field localization with significantly enhanced CD.

## 4. Discussion

The transmittance and CD value showed that the bilayer crescent chiral metasurface depends on the inter-layer distance and rotation angle (Figure 2b,d, Figure 3c,d, and Figure 4b). A pronounced CD response was observed, specifically at the rotation angle of 23° (Figure 5a,b), which will enable advanced applications in biosensing and optical filtering, by which selective interaction with CPL is important. The electric field difference for the RCP and LCP light also backs these findings. The result demonstrated that the LCP light substantially improved the electric field coupling, mainly at the edges of the metasurface’s crescent structure, in contrast to the RCP light (Figure 5c). This exclusive improvement can be attributed to the configuration of the bilayer crescent chiral metasurface, which enabled intense localized plasmonic resonance. With the increment in the rotation angle from 5° to 23°, the intensity of the electric field at the edges of the crescent increased, pointing to the significance of the rotation angle in controlling the metasurface’s optical response, as shown in Figure 5. The 2 by 2-unit cell arrangement of the crescent chiral metasurface showed a more significantly pronounced CD than that of the single-unit cell arrangement, as shown in Figure 3. This enhancement was due to the pronounced coupling between the neighboring meta-atoms in the 2 by 2-unit cell, resulting in a more complicated and robust electromagnetic response. The ensuing collective behavior in the 2 by 2-unit cell arrangement not only improved the chiroptical effects, but also allowed for more prominent and localized electric field distributions. These findings highlight the importance of the unit cell arrangement in modifying the metasurfaces’ optical properties as well as the promise for optimized designs in advanced photonic applications.

Compared with other layered chiral metasurfaces, the outcomes here showed a greater degree of control over the CD and electric field distribution by adjusting the rotation angle. Studies on single layer crescent chiral metasurfaces have shown similarities, whereas the bilayer crescent chiral metasurface examined here offers a higher CD and electric field couplings. The electric field enhancement in the single crescent structure is confined at the tip of the crescent due to the sharp curvature [39]. In contrast, our design facilitates strong coupling in between the layers as well as tuning of the chiroptical response by the extra rotational degree of freedom.

The strong CD and selective electric field enhancement of the bilayer crescent chiral metasurface makes it suitable for a range of optical applications. In optical communications, the crescent chiral metasurfaces’ capability to control polarization can enhance the signal integrity and decrease losses. In sensors and optical filters, the distinct response to the RCP and LCP light facilitates a selective response to specific polarizations, which is useful to detect chiroptical molecules. Moreover, in nonlinear optics, nonlinear effects such as harmonic generations can be boosted by the improved interaction with circular polarizations in the NIR, unveiling new avenues in the design of photonic devices.

## 5. Conclusions

This research examined the chiroptical properties of bilayer crescent chiral metasurfaces, centering on the analysis of the CD and electric field distributions across different parameters such as rotation angle, inter-layer distance, and meta-atom thickness in the NIR. We showed that when using an optimized bilayer crescent chiral metasurface, a pronounced chiroptical response could be obtained. It was demonstrated that these chiral metasurfaces exhibited a significant CD, which was dependent on the geometric parameters. Our results showed that the maximum CD was obtained at a rotation angle of 23°, inter-layer distance of 50 nm, and meta-atom thickness of 100 nm, emphasizing the potential to tailor the chiroptical response precisely by changing the geometric parameters of the metasurface. The electric field distribution enhancement difference to the LCP and RCP light of the crescent metasurface was clearly observed and computed. This selective improvement was more pronounced for the LCP, where the electric field intensity increased with rotational adjustments, highlighting the importance of the geometric parameters in optimizing the chiroptical response. It was also shown that the 2 by 2-unit cell arrangement, in comparison with the single-unit cell, exhibited a significantly increased CD due to increased interactions between neighboring cells, highlighting the importance of the unit cell arrangement in optimizing the metasurface’s optical properties for advanced photonic applications. This study contributes to a deeper understanding of the chiroptical response and its application in modern optical devices. By showing the ability to improve and control specific optical properties, our research paves the way for the development of sophisticated optical devices for sensing, optical filtering, and telecommunications. Future studies should focus on integrating these chiral metasurfaces with other photonic systems and analyzing the impact of changing the composition of the material on the observed phenomena. The potential for applying these findings in practical applications, from advanced biosensing to more efficient optical communication systems, are important and warrant additional investigation.

## Data Availability

Data are contained within the article.
